# The Quality of Sputum Specimens as a Predictor of Isolated Bacteria From Patients With Lower Respiratory Tract Infections at a Tertiary Referral Hospital, Denpasar, Bali-Indonesia

**DOI:** 10.3389/fmed.2019.00064

**Published:** 2019-04-05

**Authors:** Nyoman Sri Budayanti, Kadek Suryawan, Ida Sri Iswari, Dewa Made Sukrama

**Affiliations:** Microbiology Department, Faculty of Medicine, Udayana University, Bali, Indonesia

**Keywords:** sputum quality, Gram staining, Pathogenic bacteria, non-pathogenic bacteria, Murray Washington, antibiotic therapy

## Abstract

Sputum quality is crucial in finding infectious bacteria that will be used to guide definitive antibiotic therapy. Errors in reporting isolated bacteria will affect the rate of patients' morbidity, mortality, and increase patient care costs. This study aims to find out the relationship between sputum quality and isolated bacteria at a Tertiary Referral Hospital, Denpasar, Bali-Indonesia. The study was conducted for 6 months in the Sanglah Hospital Clinical Microbiology laboratory. There were 726 sputum specimens examined and categorized based on Murray Washington criteria. After Gram examination, all specimens were inoculated on aerobic culture media. We classified 41.4% of poor-quality sputum specimens, and non-pathogenic bacteria were isolated from 70.2% of that specimen dominated by *Streptococcus mitis* (42.53%). Whereas, isolated pathogens were obtained from 54.4% of good-quality sputum specimens dominated by *Klebsiella pneumonia* (30.86%). Statistical analyses showed that there is a relationship between isolated bacteria and the sputum quality (OR = 3.844; *p* < 0.001). Good-quality sputum is 3.8 times more likely to isolate pathogenic bacteria than poor-quality sputum. In the Pearson Chi-Square test, the likelihood of isolating pathogenic bacteria from good-quality specimens was significant too (*p* < 0.001). The results of this study indicate that poor-quality sputum specimens are still found. Therefore, the capacity of good sputum collection must be improved. Supervision of the application of standard sputum culture operational procedures must be more rigorously carried out.

## Introduction

Antimicrobial resistant (AMR) is a global problem that threatens public health causing a significant increase in morbidity and mortality across the world (1–3). The WHO surveillance report in 2014 shows that globally, 3.6% of new TB cases and 20.2% of treated cases were estimated to carry multidrug-resistant TB (MDR-TB), mostly in Eastern Europe and Central Asia with only about 48% were cured and even worst among extensively drug-resistant cases (XDR-TB). AMR also burdens the economy of any nation (4). One of the data estimated that US $ 21 to $ 34 billion dollars, accompanied by a significant increase in hospital stay, are the cost of AMR spent by the US health system (4). Although there was a decrease in inappropriate use of antibiotics based on the 2012 surveillance data in Indonesia, the inappropriate use of antibiotics in hospitals was still very high (42%). Besides, the prevalence of multidrug-resistant (MDR) bacteria increased, such as Extended-spectrum Beta-Lactamase (ESBL) *K. pneumoniae* (58%) and ESBL *E coli* (52%) and MRSA (24%) (5). The abuse of antibiotics on both quantity and quality is suspected as the major trigger for the emergence of AMR (6, 7). The general use of antibiotics should be for empirical and definitive therapies (8). Both therapies must be determined by the culture result, which is highly dependent on the quality of the specimen examined (9–11). Poor-quality specimens cause misidentification of the type of bacteria and errors in determining antibiotics for sensitivity testing (12, 13).

Sputum is one of the most frequent specimens received in a clinical microbiology laboratory at the hospital (14). Good-quality sputum will help on selecting the right antibiotics so that it would reduce and prevent the occurrence of AMR. However, sputum is also a specimen with a high rate of contamination from normal flora during the collection (15–19). This examination also could provide a prediction of isolated pathogenic bacteria, which is important information to facilitate the administration of empirical antibiotic therapy. Microscopic examination of sputum prior to the culture procedures is necessary (18, 20–22).

This study aims to investigate the relation between the quality of sputum and types of isolated bacteria in a tertiary Sanglah referral hospital in Bali. Sanglah hospital is the third and the biggest tertiary referral hospital in Bali province with a capacity of 750 beds. Operating as the highest level of a referral hospital, Sanglah Hospital commonly has to treat patients with critical condition or those who referred from other hospitals. The knowledge would help clinicians to estimate the type of isolated bacteria and help to determine the antibiotics therapy.

## Materials and Methods

Ethical clearance and permission to utilize data from Register Book of Microbiology Department of Sanglah Referral Hospital has been granted by Chief Executive Officer of Sanglah Hospital, No. KP. 03.02/X/V.2.1.1/8154/2018, dated February 28, 2018. Patient or patient's family informed consent had been made available prior to sample collection. Sanglah Hospital is a national standardized hospital, so all biosecurity measures are in place. All work has been done in biosecurity level-2 containment.

A retrospective cross-sectional study was conducted in the Laboratory of Clinical Microbiology, Sanglah Hospital, Denpasar from January to June 2018. The study population was all sputum specimen received by the lab and requested for sputum culture. The samples were sputum specimens from patients with Lower Respiratory Tract Infections (LRTI) and had been through Gram staining and sputum culture test. The diagnosis of LRTI was based on the medical record diagnosis made by the Pulmonologist Department of Sanglah Hospital. The specimens were assessed for the microscopic characterization by Gram staining and grown in Blood, McConkey, and Chocolate agar. The quality of sputum specimens was determined based on Murray-Washington criteria (23), as previously applied (24, 25). The good-quality specimens were those with ≤10 squamous epithelium and >25 leukocytes. Otherwise, it was judged as the poor-quality specimen.

The specimens were cultured and incubated 18 until 24 h at 37°C. Media with no viable growth stated as sterile, while those with the growth of more than two colonies were classified as normal flora (26). Dominant colony and suspected as the pathogen was taken for further identification and tested for antibiotic sensitivity using semi-automatic VITEK2 COMPACT (bioMeriux).

The odds ratio and Pearson Chi-Square test were calculated using Statistical Package for the Social Sciences (SPSS) version 22.

## Results

During the study period, 726 sputum specimens from LRTI patients were accepted by the Laboratory of Clinical Microbiology, Sanglah Hospital, Denpasar, Bali-Indonesia. The percentage of collected sputum specimen based on its quality from January to June 2018 is presented in Table 1. We found that the monthly average of poor-quality sputum during the study was 41.4%. The highest number was found in May (43%), while in January was the lowest (39.59%).

**Table 1:** Percentage of collected sputum specimen based on its quality from January to June 2018 in Tertiary Sanglah Referral Hospital in Bali, Indonesia.

**Sputum quality**	**January**	**February**	**March**	**April**	**May**	**June**
Good (%)	60.42	58.14	57.36	59.33	57	59.37
Poor (%)	39.58	41.86	42.64	40.67	43	40.63

**Figure 1:**
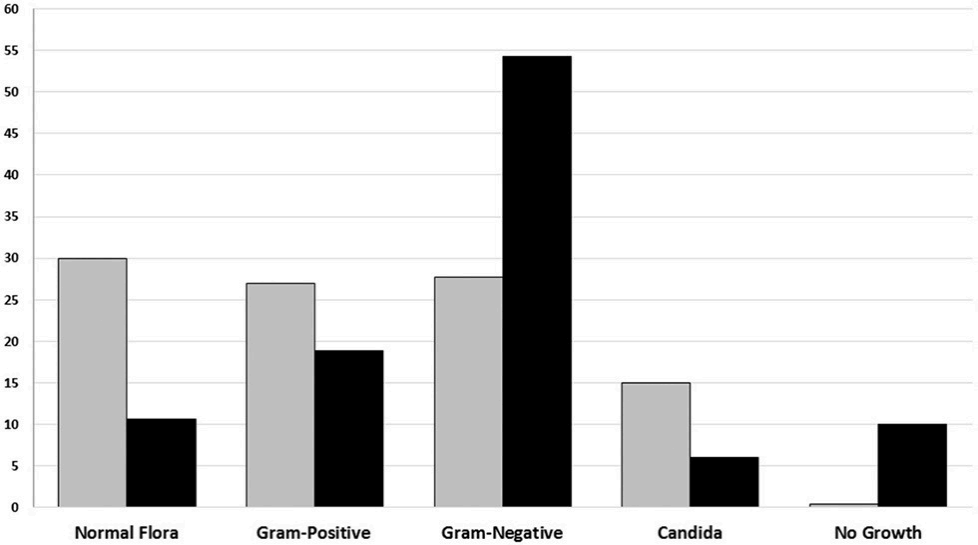
The percentage of Groups of microorganism based on sputum culture result from January to June 2018 in Tertiary Sanglah Referral Hospital in Bali, Indonesia. Gray and filled boxes are poor and good-quality sputum, respectively.

Identification based on virulence potency of isolated bacteria showed that 70.2% of the isolates from poor-quality sputum were non-pathogenic bacteria, while in good-quality sputum was 35.6%. Moreover, the percentage of no growth from good-quality sputum was higher than poor-quality sputum. There were 48 out of 726 (6.6%) of the specimens showed no viable growth on the media and those were collected from the endotracheal tube from patients with ventilator-associated pneumonia (VAP) (Table 2). Based on identification results, we found that most of the bacteria isolated from good-quality sputum were Gram-negative; while Gram-positive, normal flora and *Candida* spp. dominated the isolates from poor-quality sputum (Figure 1). Statistical analyses showed that there is a relationship between isolated bacteria and the sputum quality (OR = 3.844; *p* < 0.001). Besides, the likelihood of isolating pathogenic bacteria from good-quality specimens was significant (*p* < 0.001).

**Table 2:** Distribution of total isolated microorganism from good and poor-quality sputum specimen from January to June 2018 in Tertiary Sanglah Referral Hospital in Bali, Indonesia.

**Sputum quality**	**Type of microorganism**						**Total**	
	**Non-pathogen**		**Pathogen**		**No growth**			
	***n***	**%**	***n***	**%**	***n***	**%**	***n***	**%**
Poor	214	70.2	85	27.9	6	1.96	305	42
Good	150	35.6	229	54.4	42	9.97	421	58
Total	364		314		48		726	100

*The OR of detecting non-pathogen and pathogen in poor and good specimen was 3.844 (p < 0.001); The p-value of the likelihood of isolating pathogenic bacteria from good-quality specimens in Pearson Chi-Square test was 0.00*.

### Gram-Positive Bacteria

**Figure 2:**
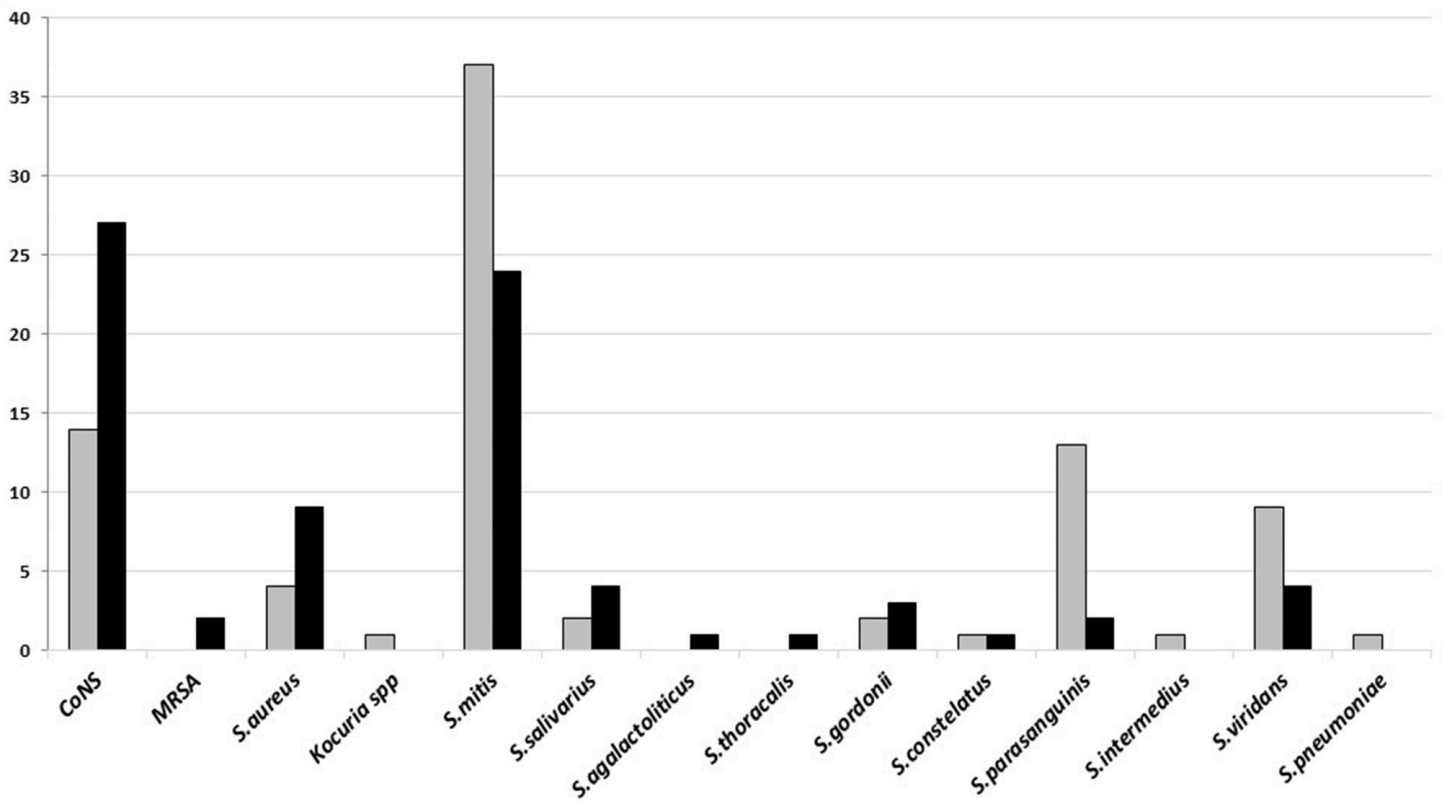
The number of Gram-positive bacteria isolated on sputum culture from January to June 2018 in Tertiary Sanglah Referral Hospital in Bali, Indonesia. Gray and filled boxes are poor and good-quality sputum, respectively.

The dominant Gram-positive bacteria was *Streptococcus mitis* and *coagulase-negative staphylococci* (CoNS) from poor and good-quality sputum, respectively. Both of the bacteria are the normal flora and sometimes found as the opportunistic bacteria in the upper respiratory tract (Figure 2).

### Gram-Negative Bacteria

**Figure 3:**
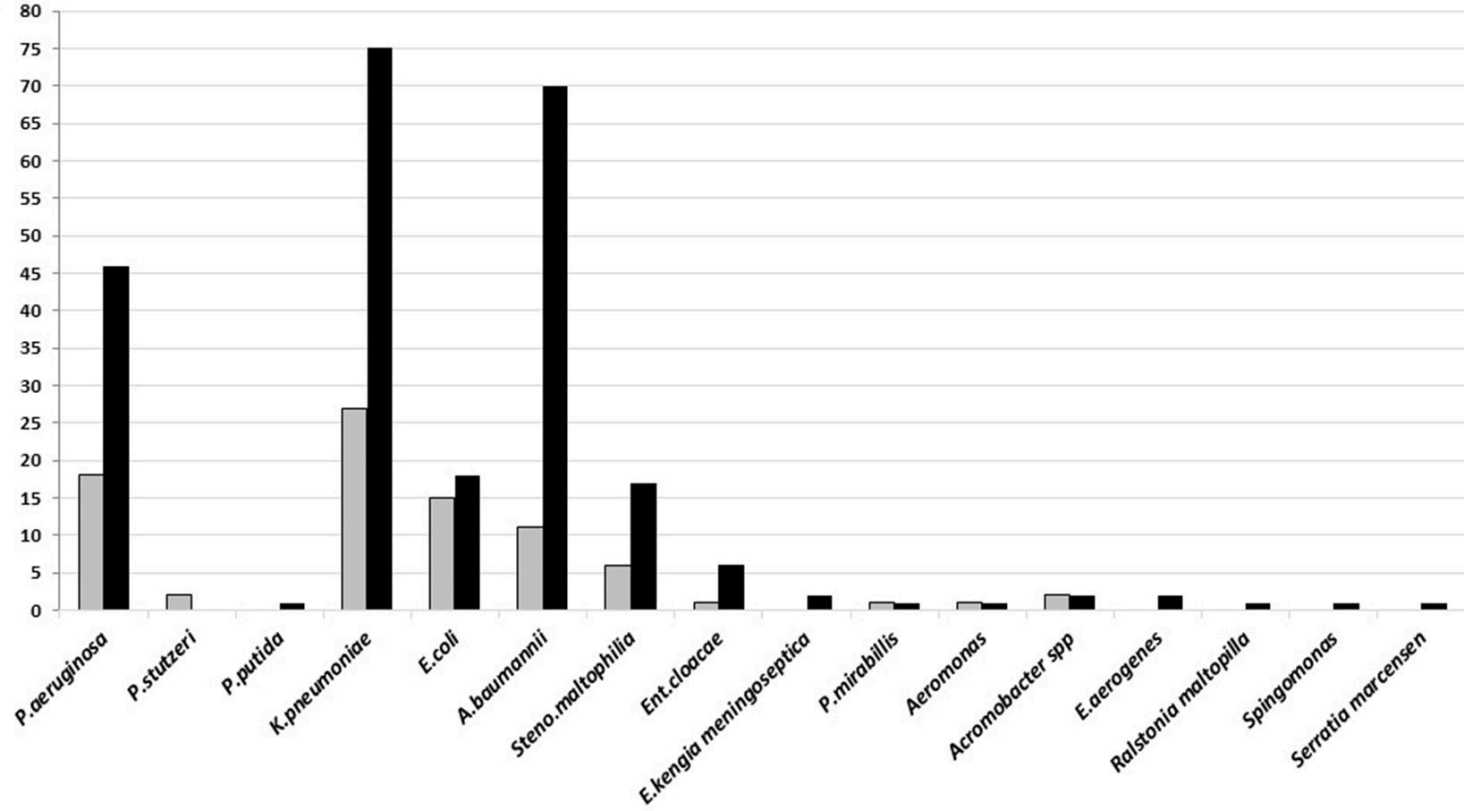
The number of Gram-negative bacteria isolated on sputum culture from January to June 2018 in Tertiary Sanglah Referral Hospital in Bali, Indonesia. Gray and filled boxes are poor and good-quality sputum, respectively.

Gram-negative bacteria, *Klebsiella pneumoniae, Acinetobacter baumannii*, and *Pseudomonas aeruginosa*, were isolated from both types of specimen, however, most of these bacteria were found in good-quality sputum. All of these dominant bacteria are known as the common cause of HAP and late VAP (Figure 3).

### Yeasts (*Candida* spp.)

The number of *Candida albican* identified in poor and good-quality sputum was 19 and 14 isolates, while other species was 25 and 16 isolate, respectively (data not shown).

## Discussion

Monthly assessment on sputum quality showed that, on average, 41% of the specimens were categorized as the poor-quality sputum. Meanwhile, an acceptable rate of poor-quality sputum on a monthly basis is 25% of the total sputum specimens (27). If the percentage of poor-quality specimens exceeds the threshold limit for three consecutive months, evaluation and specimen collection re-training must be carried out. The results of this observation indicate that there was no supervision and control over the sputum quality received in the laboratory.

The results showed that there was a significant difference in types of pathogenic and non-pathogenic bacteria in both categories of the sputum specimen which are in accordance with the results obtained by another study (26). However, this study found that the proportion of poor-quality sputum reached almost half of all received specimens. Wasting of laboratory operations such as human resources and reagents will occur if poor-quality specimens proceeded to bacterial identification procedures. Another study rejected sputum specimens since the beginning of the microscopic characterization when poor-quality specimens were found so that the identification process was discontinued (28).

No viable growth was mainly found in the good-quality sputum. This can be caused by the effect of the antibiotics consumed by the patient before specimen collection. As the highest referral hospital, Sanglah Hospital commonly treats referred and severe patients, so that most of them are on antibiotics treatment. For this reason, the ability of clinical microbiologists to collaborate with clinicians is very necessary. A clinical microbiologist should not only work in the laboratory but must go with the clinician to see and discuss the patient's condition. At present, in Indonesia, there are still many clinical microbiologists who are reluctant to collaborate with clinicians to discuss problems. Sometimes the expertise of the examination is only based on the patient's request sheet. On the other hand, many clinicians still consider clinical microbiology only working in the laboratory. This leads to worsening the use of antibiotics in the hospital so that the threat of AMR will increase followed by a high rate of morbidity and mortality.

No viable growth can happen because of virus or fungi as the agents of LRTI in the patients (29–31). As the ratio of white cells and epithelial cells was used to determine the quality of the sputum, the sputum from patients with the infection had an increase in the number of white cells in spite of infection from other pathogens.

Many CoNS were cultured from the good-quality sputum, while isolates of *Streptococcus mitis* were mostly from poor-quality sputum. Both bacteria are upper respiratory tract flora. CoNS, known as an opportunistic pathogen and dominated colonies in growth media, is sometimes considered as the cause of infection, particularly when it is found from good-quality sputum specimen. Some medical conditions such as early VAP and aspiration pneumonia may turn that bacteria into a pathogen. Group of commonly found Gram-negative bacteria in hospital settings, *K. pneumoniae, A. baumannii*, and *P. aeruginosa*, were dominant in both good and poor-quality sputum culture, although the number of that bacteria was isolated more from the good-quality sputum. These results are different from another study which found that the majority of *Streptococcus pneumoniae* was isolated from patient's sputum (32). It may be caused by sputum specimens that were collected from patients with HAP or from those with long antibiotics therapy. The high variation on type and number of isolated *Candida* spp. indicates of prolonged use of antibiotics. Culture examination before giving empirical or changing antibiotics treatment is not yet common conduct of the clinicians in developing countries. It is frequently found in hospitals, in Indonesia, to conduct culture examination only if the patient has no sign of improvement from an infection and already treated using various antibiotics.

The major limitations of this paper was that time points the sample were taken and the detailed history of antibiotic therapy was not available in the request form, as well as the antibiotics administration without culture. This is a major problem in developing country (33, 34).

Some factors impeded good practices of clinical microbiology in developing countries, particularly in Indonesia. These factors include no special education for microbiology laboratory analysts, a limited number of clinical microbiologists compared to the total area and population of Indonesia, lack of awareness among clinicians on the importance of culture examination to guide definitive therapy, and reluctance mindset to collaborate between clinicians and clinical microbiologists in infection treatment. All of these factors must be addressed immediately to allow the best practices for clinical microbiology for being carried out. Not only in terms of human resources, but laboratory facilities and infrastructures with qualified human resources are needed. Training and dissemination of the roles of clinical microbiology in handling infection cases, especially the selection of antibiotics, must be immediately implemented to reduce the increasing rate of AMR in Indonesia.

## Author Contributions

NB and DS designed and supervised the study. NB interpreted the data and prepared the manuscript with support from DS. KS and II was organized the data acquisition including sampling and sample preparation as well as data analysis. All authors gave the approval of the final version manuscript.

### Conflict of Interest Statement

The authors declare that the research was conducted in the absence of any commercial or financial relationships that could be construed as a potential conflict of interest.
